# Use of AI to Predict and Support Medication Adherence in Patients With Breast Cancer: Systematic Review

**DOI:** 10.2196/80128

**Published:** 2026-04-21

**Authors:** Massimo Pezzolato, Viktorya Voskanyan, Ilaria Cutica, Chiara Marzorati, Gabriella Pravettoni

**Affiliations:** 1 Applied Research Division for Cognitive and Psychological Science, European Institute of Oncology, Via Ripamonti, 435, Milan, Italy, 39 029437209; 2 Department of Oncology and Hemato-oncology, University of Milan, Milan, Italy

**Keywords:** breast cancer, medication adherence, artificial intelligence, predictive modelling, endocrine therapy, oral anticancer medications

## Abstract

**Background:**

Oral medications are commonly used in the treatment of breast cancer (BC), despite high rates of nonadherence. As adherence is fundamental for optimal treatment, finding ways to effectively improve it is important. Artificial intelligence (AI) is being widely applied to health care.

**Objective:**

This review aims to offer an overview of the contribution of AI to medication nonadherence among patients with BC, suggesting future research directions, and highlighting existing gaps.

**Methods:**

Four databases (PubMed, Embase, Scopus, and Web of Science) were searched. Studies were eligible if they used AI to predict, monitor, or support medication adherence in patients with BC, were published in English, and had full text available. Reviews, conference abstracts, editorials, and studies with mixed samples were excluded. This review was conducted following PRISMA (Preferred Reporting Items for Systematic Reviews and Meta-Analyses) guidelines and was registered in PROSPERO (International Prospective Register of Systematic Reviews) database (CRD42024587020). The risk of bias was evaluated using the PROBAST (Prediction Model Risk of Bias Assessment Tool) and the Downs and Black’s methodological quality scale.

**Results:**

Ten studies were included in this review. Most of them (k=9) focused on developing machine learning models to predict medication nonadherence. These studies used a range of techniques, including logistic regression, artificial neural networks, and random forests. Model performance varied widely, with area under the receiver operating characteristic curve values ranging from 0.61 to 1.00. Predictors of nonadherence were clustered into 4 groups: clinical, disease, and treatment-related factors (eg, side effects and comorbidities); behavioral factors (eg, prior nonadherence); psychosocial factors (eg, quality of life and self-efficacy); and sociodemographic factors (eg, age and income). The only intervention study identified evaluated an AI-based chatbot and reported promising results, showing a 20% increase in adherence among participants who engaged with its reminder feature. All included studies were at high risk of bias, mainly due to the absence of model calibration or insufficient reporting, and their findings should therefore be interpreted with caution. A further limitation was the lack of attention to implementation: predictive accuracy alone is insufficient, and future work must also address actionability, safety, and cost-effectiveness to enable real clinical use. Progress in this area will require coordinated efforts among researchers, developers, clinicians, and policymakers to support the responsible development and implementation of these tools into routine care.

**Conclusions:**

To our knowledge, this is the first systematic review of AI applications specifically targeting medication adherence in BC. It focuses on both predictive and interventional studies, mapping current AI applications within this specific clinical context. The findings highlight gaps in the implementation phase and emphasize the need for future research integrating a coordinated, multidisciplinary approach involving researchers, AI specialists, policymakers, and health care teams.

## Introduction

The use of oral antineoplastic agents has rapidly increased in cancer management over the last few decades, improving overall survival rates and offering the convenience of self-administered treatment [1-3]. While oral agents can enhance cancer care by giving patients greater autonomy and control over their treatment, they also demand active participation, adding complexity to an already demanding routine. Following a cancer diagnosis, patients must manage frequent hospital visits, lifestyle adjustments such as following a healthy diet and staying physically active, and now also adhering rigorously to an oral medication regimen, introducing an additional layer of responsibility [4,5]. Often, this occurs at a time when patients are psychologically and physically drained, having to deal with fear, worries, anxiety, and treatment side effects, all of which contribute to reduced quality of life [6] and further complicate the challenge of optimal medication adherence.

Medication adherence refers to the extent to which patients take their medications as prescribed by the health care provider, including the correct timing, frequency, and dosage [7]. The phenomenon of nonadherence is common across different patient populations, with estimates suggesting that approximately 50% of adult patients fail to achieve optimal adherence [8,9].

Adherence among patients with breast cancer (BC) has been reported to range from 33.3% to 88.6%, with an average decrease of 25% from the first to the fifth year of treatment [10]. Although these estimates span a wide range, they highlight that nonadherence is a significant concern in this clinical population. This is particularly alarming given its association with higher mortality rates, poorer clinical outcomes, and increased health care costs [11,12].

A wide range of factors may influence patients’ medication-taking behaviors and contribute to nonadherence, including clinical factors (related to the disease and its treatment), psychosocial and behavioral aspects, sociodemographic characteristics (eg, age, geographic area, and income), and health care system factors (eg, the quality of the patient-provider relationship) [9,13]. Among these factors, commonly reported influences on treatment adherence in patients with BC include the presence of side effects associated with oral antineoplastic agents (eg, joint pain, hot flushes, depression, and weight gain), patients’ perceptions of the necessity of treatment or concerns about it, poor quality of the patient-provider relationship, and a lack of social, practical, or emotional support [13]. This overview highlights that adherence is a complex phenomenon, influenced by a diverse set of interrelated and multifaceted factors.

Given the scale of the problem and its negative consequences, improving adherence is essential. However, the various interventions proposed so far have shown only limited effectiveness, and the need for innovative solutions to predict and support medication adherence is still pressing [5,14-17]. Artificial intelligence (AI)–driven technologies, considering their significant advancements and efficiency across various domains [18,19], have the potential to play a key role in bridging this gap.

AI is an umbrella term commonly used to refer to those technologies that simulate complex human skills such as learning, creativity, decision-making, and problem-solving [20-22]. AI can be further characterized into subsets such as machine learning (ML), natural language processing (NLP), and generative artificial intelligence (GenAI) [20].

ML refers to a class of algorithms that are trained on data to automatically learn and improve their performance over time. These algorithms are typically easy to interpret, perform effectively on small to medium-sized datasets, and require relatively short implementation times. Deep learning (DL), a subset of ML, provides more advanced capacities to handle large and complex datasets. However, DL models typically require greater computational resources and involve longer training times due to their multilayered and complex architectures. Moreover, these models are more challenging to interpret compared to traditional ML approaches. Despite these constraints, DL has demonstrated notable results in tasks such as image recognition, cancer subtype classification, and clinical decision-making [23,24]. A particularly relevant application of ML and DL is predictive modeling, a process in which computational analyses are used to generate predictions about the occurrence of specific events or outcomes. ML-powered predictive models have been used in medicine and health care with promising results in areas such as clinical decision-making, disease progression prediction, and the identification of high-risk patients for early intervention [25].

NLP comprises a set of techniques used to process, understand, and sometimes generate human language [20]. NLP models are widely used in health care for tasks involving the processing of speech or text, such as sentiment analysis, screening of electronic health records, computer-aided diagnosis, and support for clinical decision-making [20,26,27].

GenAI is a subset of AI that leverages ML models and NLP techniques to generate content in various formats, such as text, speech, and images. Conversational agents (also known as chatbots) are a widely adopted application of GenAI, leveraging ML and NLP to engage in dialogue, answer questions, provide suggestions, and perform a variety of tasks. In the health care domain, they have been used to deliver patient education, support treatment and monitoring, promote medication adherence, facilitate behavior change interventions, and assist in diagnosis [20,28].

Given its significant impact across various health care domains, such as screening, diagnostics, symptom management, personalized risk prediction, clinical decision-making, and patient engagement [29-32], AI may also represent a promising tool for addressing medication adherence in patients with BC. In this context, AI can contribute to 2 distinct yet interconnected aspects of the problem. First, it may directly support patients in sustaining adequate adherence, for example, when embedded in tools such as chatbots or eHealth platforms that can provide information, reminders, and tailored interventions. Second, AI can analyze large and complex datasets more efficiently than traditional statistical approaches [33], enabling the development of predictive models that identify patients at higher risk of nonadherence who may require timely, targeted support. Although this second contribution does not act directly on patients’ behavior, it remains highly relevant. Many health care systems operate under significant resource constraints, making it unrealistic to provide adherence-supporting interventions to all patients with BC. Prioritizing those most likely to benefit is therefore crucial. Thus, this systematic review aims to offer a comprehensive overview of AI-based approaches used to address medication nonadherence among patients with BC, either by predicting the risk of nonadherence or by monitoring and directly supporting adherence. To our knowledge, this is the first systematic review covering the wide range of AI capabilities addressing nonadherence in this population. Furthermore, the review offers suggestions for future research directions, highlighting open challenges and ethical considerations.

## Methods

### Study Design

This systematic review was conducted following the PRISMA (Preferred Reporting Items for Systematic Literature Reviews and Meta‐Analysis) guidelines [34]. The PRISMA checklist is presented in Checklist 1. This review was registered in the PROSPERO (International Prospective Register of Systematic Reviews; CRD42024587020) after conducting the initial database search. A protocol for the review was not prepared.

A meta-analysis was not performed due to substantial heterogeneity, specifically a high degree of clinical and methodological diversity across studies, including differences in study design, AI techniques, and in the operationalization and measurement of medication adherence. All these factors made the statistical pooling inappropriate and potentially misleading.

### Search Strategy

#### Overview

A search strategy was developed and reported in accordance with the PRISMA-S (Preferred Reporting Items for Systematic Reviews and Meta-Analyses literature search extension) guidelines [35]. The search was conducted by 2 authors (MP and VV) in the following databases: Scopus (Elsevier), PubMed (National Library of Medicine), Embase (Elsevier), and Web of Science Core Collection (Clarivate). No language, publication date, or other search restrictions were applied.

The keywords revolved around three core review terms: (1) adherence, (2) BC, and (3) AI (see Multimedia Appendix 1 for the complete search strategy). Studies published until February 4, 2026, were captured.

#### Inclusion and Exclusion Criteria

Studies were considered eligible when (1) they reported on any AI technology (eg, ML, DL, NLP, and GenAI) being used to predict, monitor, or support oral medication adherence, (2) they involved a patient with BC sample, (3) the paper was written in English, and (4) the full text was available online. Reviews, conference abstracts, and editorials were excluded. Additionally, studies with mixed populations where it was impossible to separate the results of patients with BC were excluded.

#### Screening

The screening was organized in Rayyan software: a web and mobile tool for reviews [36], which was also used to identify and remove duplicate records before screening. Both title or abstract and full-text screening were conducted by 2 researchers (MP and VV) in “blind mode” (ie, researchers were unaware of each other’s decisions). After the initial screening (title or abstract), MP and VV read the full text of the retrieved papers that met the inclusion criteria to confirm their relevance. Discrepancies in the judgments during both title or abstract and full-text screening were sent to a third author (IC), and disagreements were settled by discussion until reaching a consensus.

#### Data Extraction and Summary

Data were extracted using a shared, standardized table developed jointly by MP and VV at the outset of the review, which was applied consistently across all studies. For each included paper, relevant characteristics and results were extracted and inserted into the spreadsheet. The extracted data were authors, title, year of publication, country, study design, aim, sample size, AI subtypes and algorithms, adherence measure, and results.

For studies describing the development of ML predictive models, information on predictors, model performance metrics, and types of validation was also extracted. Performance was summarized using discrimination measures, including the area under the receiver operating characteristic curve (AUC), *F*_1_-score, concordance index (C-index), and accuracy.

In this paper, because the 2 types of evidence differ substantially, separate Results and Discussion sections were developed to present findings from studies describing the development of ML predictive models and from the single intervention study identified.

The data were extracted by 2 authors (MP and VV) and checked by 2 researchers (IC and CM). Formal interrater agreement was not assessed.

### Risk of Bias Assessment

For studies describing the development of ML predictive models, the risk of bias was assessed using the PROBAST (Prediction Model Risk of Bias Assessment Tool) [37]. PROBAST evaluates the risk of bias in predictive modeling studies through 20 signaling questions distributed across 4 domains: participants, predictors, outcome, and analysis. In addition, it assesses concerns regarding the applicability of the predictive models in the domains of participants, predictors, and outcome.

Conversely, the risk of bias in the study describing the implementation of an intervention to support patients with BC was evaluated with Downs and Black’s methodological quality scale [38]. This scale comprises 27 items and provides a score on 5 different methodological quality dimensions: reporting bias, external validity, internal validity, confounding - selection bias, and statistical power.

The risk of bias assessment was conducted by 2 authors (MP and VV), and disagreements were resolved by discussion until reaching a consensus.

## Results

### Summary of Study Characteristics

**Figure 1.**
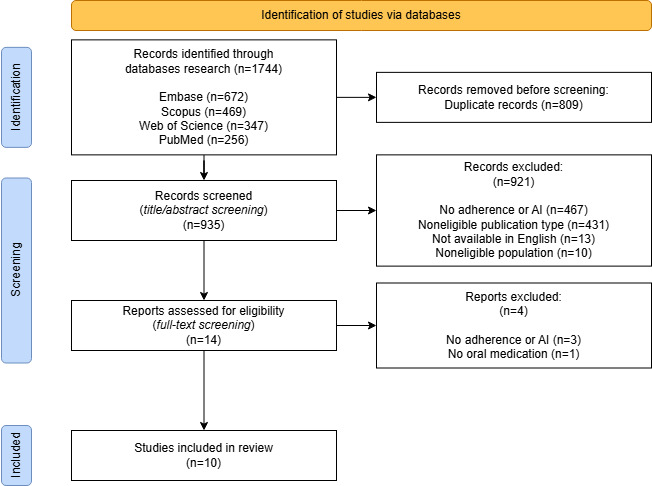
PRISMA flowchart illustrating the selection of studies included in this review. PRISMA: Preferred Reporting Items for Systematic Reviews and Meta-Analyses.

The search returned a total of 1744 papers. After duplicate removal and screening of titles and abstracts, 14 studies were considered eligible. After screening the full text, 10 of them met our inclusion criteria (see Figure 1 for a detailed flowchart of the screening process).

The included studies were published between 2019 and 2025. Most of them were conducted in the United States (k=5) and Europe (France: k=3; Italy: k=1), with 1 study carried out in Taiwan. Both early-stage BC stages (k=5) and metastatic patients (k=2) were represented in different studies, and 3 studies did not report patients’ cancer stages. The sample sizes of the studies ranged from 18 to 229,695.

Adherence was measured using administrative health data or medical records (k=4), self-report measures (k=3), and medication event monitoring systems (k=3).

A total of 9 of the included studies described the development of ML predictive models (7 retrospective cohort studies, 1 prospective study, and 1 randomized controlled trial protocol), while only 1 prospective study described the implementation of an AI-powered intervention to support oral medication adherence.

The key characteristics of the included studies are summarized in Table 1, while Table 2 provides an overview of the AI-based predictive models and adherence support intervention.

**Table 1.** Key characteristics of the included studies, including author, year, title, country, design, study aim, sample size, and adherence measures.

Author	Year	Title	Country	Design	Aim	Sample size	Adherence measure
Balazard et al [39]	2023	Adjuvant endocrine therapy uptake, toxicity, quality of life, and prediction of early discontinuation.	France	Retrospective cohort study	To develop a prediction model of early ET^a^ discontinuation.	BC^b^, stage I-III (descriptive analyses, n=6488; model development, n=5282; training set, n=4225; validation set, n=1057)	Patients’ self-declarations recorded in electronic case report forms.
Chaix et al [40]	2019	When chatbots meet patients: one-year prospective study of conversations between patients with breast cancer and a chatbot.	France	Prospective study	To evaluate 1 year of conversations between patients with BC and a chatbot.	BC, stage N/A^c^ (n=958; adherence measurement, n=33)	Answers to the chatbot’s reminder messages were used to measure adherence rates.
Kaur et al [41]	2025	A computational framework for longitudinal medication adherence prediction in breast cancer survivors: a social cognitive theory based approach.	United States	Retrospective cohort study	To predict daily and weekly adherence by integrating static and dynamic factors.	BC, stage I-III (n=32)	Medication event monitoring system.
Kaur et al [42]	2021	Theory-guided randomized neural networks for decoding medication-taking behavior.	United States	Retrospective cohort study	To predict daily medication-taking behavior over 3 time points (baseline, 4 months, and 8 months).	BC, stage I-III (n=32)	Medication event monitoring system.
Kuo et al [43]	2022	Using data mining technology to predict medication-taking behaviour in women with breast cancer: a retrospective study.	Taiwan	Retrospective cohort study	To predict long-term AHT^d^ adherence and persistence.	BC, stage n.a. (n=385)	Clinical registry and medical records (MPR^e^>80%).
Masiero et al [44]	2023	A machine learning model to predict patients’ adherence behavior and a decision support system for patients with metastatic breast cancer: protocol for a randomized controlled trial.	Italy	RCT^f^ protocol	To evaluate a decision support system and an ML^g^ predictive model, and to refine it with a set of new variables.	mBC^h^ (RCT, n=100; retrospective data for the predictive model, n=2750)	Weekly medication diaries and self-report measures.
Rattsev et al [45]	2024	Incorporation of emergent symptoms and genetic covariates improves prediction of aromatase inhibitor therapy discontinuation.	United States	Retrospective cohort study	To build a survival ML model for the prediction of aromatase inhibitor discontinuation and to explore how the predictive value of various risk factors changes over time.	BC, stage 0-III (n=181)	Medical records (therapy discontinuation).
Rinder et al [46]	2024	Dynamic projection of medication nonpersistence and nonadherence among patients with early breast cancer.	France	Retrospective cohort study	To model persistence and adherence to oral anticancer treatment.	eBC^i^ (n=229,695)	Administrative health data (MPR>80%).
Yerrapragada et al [47]	2021	Machine learning to predict tamoxifen nonadherence among US commercially insured patients with metastatic breast cancer.	United States	Retrospective cohort study	To predict tamoxifen nonadherence in the first year after treatment initiation using ML algorithms to identify predictive factors, and to evaluate administrative data for the development of a nonadherence screening tool.	mBC (n=3022)	Administrative health data (MPR>80%).
Yuan et al [48]	2025	Multimodal sensing and modeling of endocrine therapy adherence in breast cancer survivors.	United States	Prospective study	To predict daily medication adherence using multimodal data.	BC, stage I-III (descriptive statistics, n=20; analysis, n=18)	Medication event monitoring system.

a ET: endocrine therapy.

b BC: breast cancer.

c N/A.: not available.

d AHT: adjuvant hormonal therapy.

e MPR: medication possession ratio.

f RCT: randomized controlled trial.

g ML: machine learning.

h mBC: metastatic breast cancer.

i eBC: early-stage breast cancer.

**Table 2.** Summary of AI^a^-based predictive models and adherence-support interventions, detailing technologies and algorithms used, predictors, performance metrics, validation approaches, intervention features, and main results. Higher values of AUC^b^, accuracy, and C-index^c^ indicate better predictive performance.

Author	Year	AI technologies/algorithms	Predictors	Performance metrics	Validation	Intervention	Results
Balazard et al [39]	2023	ML^d^/L2-penalized Cox regression, gradient boosted trees with a Cox partial likelihood	Discontinuation of ET^e^, QoL^f^, physical functioning, role functioning, social functioning, emotional functioning, cognitive functioning scores, mastectomy and radiotherapy, fatigue, pain, dyspnea, insomnia, systemic therapy side effects, breast symptoms, and arm symptoms.	C-index (0.60‐0.64).	Internal (nested cross-validation and held-out validation set)	N/A^g^.	Patients who have already discontinued an ET after primary treatment were more likely to discontinue ET definitively; higher QoL, physical functioning, role functioning, social functioning, emotional functioning, cognitive functioning scores, and receiving mastectomy and radiotherapy were associated with lower rates of early ET discontinuation; fatigue, pain, dyspnea, insomnia, systemic therapy side effects, breast symptoms, and arm symptoms were associated with a higher rate of early ET discontinuation.
Chaix et al [40]	2019	ML/N/A	N/A.	N/A.	N/A	A chatbot that interacts with users by simulating a human conversation through text was designed to empower patients and answer their questions with personalized insights.	The overall satisfaction was 93.95% (900/958). The average compliance of patients using the medication reminder feature improved by more than 20%.
Kaur et al [41]	2025	DL^h^/long short-term memory, feed-forward neural networks, SHAP^i^	Medication-taking behavioral patterns, symptom burden, perceived susceptibility, decision regret, and medication-taking self-efficacy.	Accuracy (87.25; 76.04), precision (92.04; 75.83), recall (93.15; 85.8), and specificity (77.5; 72.3).	Internal (nested cross-validation)	N/A.	The models showed strong predictive performance, outperforming traditional ML models across all evaluated performance metrics in the daily model, and in terms of accuracy, recall, and specificity in the weekly model.
Kaur et al [42]	2021	ML/randomized neural networks	Survey data regarding BC^j^ survivorship knowledge, medication attitudes, medication self-efficacy, QoL, and other factors.	Overall accuracy and individual accuracy (0.92<OA^k^ <0.97).	N/A	N/A.	The model outperforms existing computational models in terms of prediction accuracy under conditions of randomness. It was able to predict the randomness of the subjective values and decision rules that contribute to the dynamics of patients’ medication-taking behavior.
Kuo et al [43]	2022	ML/decision tree, artificial neural networks	The top 5 influencing factors were duration of AHT^l^ discontinuation, duration of AHT use, age at diagnosis, BMI, and receipt of radiotherapy.	Overall accuracy, specificity, sensitivity, and AUC (0.90‐1.00).	N/A	N/A.	All 3 models achieved high classification performance. Multiple logistic regression was the most effective approach (accuracy: 96.37%; specificity: 96.75%; sensitivity: 96.12%).
Masiero et al [44]	2023	ML, NLP^m^/Shapley values	Sociodemographic variables, diagnosis, biochemical and medical tests, procedures and medical history, treatment programs, treatment side effects, comorbidities, and familiarities, initiation of the treatment interruption, and skipped treatment doses. The RCT^n^ will allow the addition of the following variables as predictors to the predictive model: personality traits, self-efficacy for coping with cancer, sense of coherence, pain, anxiety, depression, risk perception, and QoL.	AUC, precision, recall, sensitivity, specificity, κ, and positive and negative predictive values.	N/A	Participants in the intervention group will have access to a web-based decision support system, and an ML-based application will be used in shared decision-making sessions.	N/A.
Rattsev et al [45]	2024	ML/Cox proportional hazards, random survival forest, penalized Cox proportional hazards, gradient boosted models, and SHAP	Genetic markers, disease stage, sleep disturbance, anxiety, depression, previous taxane therapy, fatigue, age, self-reported adherence, endocrine symptoms, physical function, BMI, and joint pain.	Mean AUC (0.57‐0.66), integrated Brier score, time-dependent AUC, sensitivity, specificity, and positive predictive values.	Internal (cross-validation)	N/A.	The results indicated that integrating genomic and longitudinal features enhances the model’s predictive performance, thereby potentially facilitating the identification of patients at risk of early discontinuation.
Rinder et al [46]	2024	DL/gated-recurrent unit network, feed-forward neural network, and SHAP	Age (>70 y), past nonadherence, taking more than 1 treatment in the previous 3 months, and low income were predictors of nonpersistence. The predictors of nonadherence were similar, adding age (<50 y) and irregular intervals in treatment purchases.	AUC (0.71‐0.73).	Internal (held-out validation set)	N/A.	The model was able to estimate the risk of nonpersistence and nonadherence among French female patients with localized BC and identified that some factors (age, past behavior, and number of treatments) may be associated with the risk of nonpersistence and nonadherence.
Yerrapragada et al [47]	2021	ML/logistic regression, boosted logistic regression, random forest, feed-forward neural network models	Patient features (55 y or older at treatment start, receiving care in the South, being a surviving spouse), pretreatment procedures (lymphatic nuclear medicine, radiation oncology, arterial surgery, microbiology, and imaging) or therapy (β-blockers, antidepressants, and stimulants), and baseline comorbid diagnoses (upper respiratory disease, breathing abnormality, dorsopathy, neurotic disorders, cerebrovascular disease, abdominal pain, and genital disorders).	AUC (0.61‐0.64), *F*_1_-score (0.59‐0.62), sensitivity, and specificity.	Internal (cross-validation)	N/A.	All models had poor predictive accuracy. Logistic regression (AUC=0.64) was interpreted with 94% sensitivity (95% CI 89 to 92) and 0.31 specificity (95% CI 29 to 33). The model accurately classified adherence (negative predictive value 89%) but failed in discriminating nonadherence (positive predictive value 48%).
Yuan et al [48]	2025	DL/random forest, XGBoost^o^, light gradient boosting machine, long short-term memory, SHAP	Recent adherence history, subjective reports of mood, physical side effects, social support, context of medication-taking behaviors, physical activity, sleep, cardiovascular function, demographic data, and static psychosocial traits.	Macro-averaged balanced accuracy (0.84), macro-averaged *F*_1_-score (0.88).	Internal (nested cross-validation)	N/A.	Results indicated the feasibility of using multimodal sensing data to predict daily adherence with moderate accuracy. Models that integrated multimodal data consistently outperformed those relying on a single modality.

a AI: artificial intelligence.

b AUC: area under the receiver operating characteristic curve.

c C-index: concordance index.

d ML: machine learning.

e ET: endocrine therapy.

f QoL: quality of life.

g N/A: not available.

h DL: deep learning.

i SHAP: Shapley Additive Explanations.

j BC: breast cancer.

k OA: overall accuracy.

l AHT: adjuvant hormonal therapy.

m NLP: natural language processing.

n RCT: randomized controlled trial.

o XGBoost: Extreme Gradient Boosting.

### Predictive Models

Most of the included studies (n=9) described the development of ML predictive models [39,41-48]. By feeding ML algorithms with diverse types of patient data, these models aim to offer predictions about which patients are more likely to become nonadherent or to face issues related to medication-taking along their care journey.

Only 2 studies [39,45] developed regression models, using a numerical outcome of interest: the time from treatment initiation to discontinuation. All other studies focused on classification models, defining adherence as a categorical variable and aiming to predict which patients may experience difficulties with medication adherence.

Overall, the performance metrics (ie, AUC, C-index, *F*_1_-score, and accuracy) ranged from poor to excellent (see Table 3 for a summary of the model performance, excluding post hoc analyses).

**Table 3.** Performance metrics of the predictive models evaluated in this study, excluding post hoc analyses.

First author, year	Performance metrics
Balazard et al [39], 2023	C*^a^*=0.60‐0.64
Kaur et al [41], 2025	A^b^=87.25 (daily model); A=76.04 (weekly model)
Kaur et al [42], 2021	OA*^c^*=0.92‐0.97
Kuo et al [43], 2022	AUC^d^=0.90‐1.00
Masiero et al [44], 2023	N.a.^e^
Rattsev et al [45], 2024	AUC=0.71(6-mo); AUC=0.67 (12 mo; mean time-dependent AUC=0.65)
Rinder et al [46], 2024	AUC=0.71‐0.73
Yerrapragada et al [47], 2021	AUC=0.61‐0.64; F^f^=0.59‐0.62
Yuan et al [48], 2025	BA^g^=0.84, F=0.88^h^

a C: concordance index.

b A: accuracy.

c OA: overall accuracy.

d AUC: area under the receiver operating characteristic curve.

e N.a.: not available.

f F: *F*_1_-score.

g BA: macro-averaged balanced accuracy.

h Macroaveraged *F*_1_-score.

Several diverse ML algorithms were implemented. Balazard et al [39] evaluated the performance of 2 complementary ML models: L2-penalized Cox regression and gradient boosted trees with a Cox partial likelihood. The models showed similar performance with C-indexes ranging from 0.60 to 0.64. They were first validated internally using nested cross-validation on the training set, and the best-performing model was then evaluated on the held-out validation set.

Yerrapragada et al [47] trained and evaluated 4 models: logistic regression, boosted logistic regression, random forest, and feed-forward neural network. The models were internally validated using 10-fold cross-validation and showed poor performance with AUC ranging from 0.61 to 0.64 and *F*_1_-scores ranging from 0.59 to 0.62 [49]. The authors then applied a post hoc balancing technique, the synthetic minority oversampling technique; the evaluation after the refitting showed an improvement in the performance of the random forest model (AUC 0.93, *F*_1_-score 0.78) and the feed-forward neural network model (AUC 0.79, *F*_1_-score 0.71).

Kuo et al [43] developed an ensemble learning scheme by applying 3 ML models: multiple logistic regression, decision trees, and artificial neural networks. All models demonstrated high performance, with AUC values ranging from 0.90 to 1.00 and accuracy between 92.29% and 96.37%. However, the authors did not specify whether any validation method was applied to the models.

Kaur et al [42] designed a randomized neural network and compared its prediction performance (overall accuracy and individual accuracy) with that of a traditional neural network. The randomized neural network model outperformed the traditional one, achieving high accuracy levels (>0.92) both overall and at the individual level. Although the paper did not report any internal or external validation, the authors indicated their intention to perform leave-one-subject-out cross-validation to properly evaluate the developed model.

The same research group subsequently developed other DL models combining long short-term memory and feed-forward neural networks [41]. Specifically, they built a daily model to predict patients’ medication-taking behavior for the following day and a weekly model to predict adherence over the following week. When compared with traditional ML approaches (ie, random forest, support vector machine, XGBoost [Extreme Gradient Boosting], and logistic regression), the proposed models demonstrated superior performance. For the daily and weekly models, respectively, accuracy reached 87.25 and 76.04, precision 92.04 and 75.83, recall 93.15 and 85.8, and specificity 77.5 and 72.3. With regard to internal validation, the study adopted a nested 5-fold cross-validation approach. In addition, the contribution of individual predictors was examined using SHAP (Shapley Additive Explanations) values.

Rinder et al [46] developed a DL predictive model by combining a recurrent neural network and a feed-forward neural network. The model demonstrated satisfactory predictive performance, with an AUC of 0.71 for persistence and 0.73 for adherence. Internal validation was performed using a 60:20:20 train-validation-test split. Once again, SHAP values were analyzed to gain insights into the specific factors’ contributions to the model’s outcomes.

Masiero et al [44] developed ML predictive models currently undergoing evaluation in a randomized controlled trial involving patients with metastatic BC. The predictive models were developed by applying NLP techniques to an electronic health record dataset, and they will be further optimized by feeding newly collected data during the randomized controlled trial. The models’ predictive performance was evaluated with several metrics (AUC, precision, recall, sensitivity, specificity, κ, and positive and negative predictive values), but the results of the evaluation are not reported. The predictive models use Shapley values for interpretability, allowing the evaluation of how much each specific risk factor contributes to the given prediction.

Yuan et al [48] developed a 2-tier DL ensemble to predict daily adherence. At tier 1, long short-term memory models and tree-based regressors (ie, random forest, XGBoost, and LightGBM [light gradient boosting machine]) were trained. At tier 2, the outputs of these models were integrated using an optimized soft-voting strategy. The resulting multimodal model predicted adherence more accurately than single-modality models, achieving a balanced accuracy of 0.84 and an *F*_1_-score of 0.88. The model was validated using leave-one-participant-out cross-validation, and its predictors were interpreted using SHAP analysis.

Finally, Rattsev et al [45] developed a survival ML model to predict time-to-discontinuation of aromatase inhibitors. The predictive framework was based on 4 survival ML algorithms: Cox proportional hazards, random survival forest, penalized Cox proportional hazards, and gradient boosted models. The results indicated that incorporating genetic markers and follow-up data improved model performance; however, overall discrimination remained modest, with a mean time-dependent AUC of 0.65 and AUC values of 0.71 and 0.67 at 6 and 12 months, respectively. As in the previous studies, SHAP values were used to examine the relative importance of individual features.

### Predictors of Nonadherence

The studies describing the development of predictive models using ML identified several predictors of nonadherence. These can be categorized into four groups: (1) clinical, disease-, and treatment-related factors; (2) behavioral factors; (3) psychosocial factors; and (4) sociodemographic factors.

Among clinical, disease-related, and treatment-related factors, the presence of side effects or symptoms [39,41,45,48], complex drug regimens [46,47], comorbidities [47], and low BMI [43] were identified as predictors of nonadherence. Likewise, an increase in BMI was associated with decreased risk of discontinuation [45]. In addition, prior taxane therapy was associated with a higher risk of discontinuation, whereas specific genetic markers and more advanced disease stages were associated with a lower risk [45]. Contradictory results were identified regarding some treatment-related variables. One study found that a history of pretreatment radiotherapy or diagnostic imaging procedures was associated with higher nonadherence [47], whereas another study reported that a recent mastectomy or radiotherapy was associated with increased adherence [39].

Behavioral factors associated with nonadherence included a history of previous nonadherence [39,46], a longer time since treatment initiation, and longer durations of treatment discontinuation episodes [43]. In line with these findings, higher levels of self-reported adherence were associated with a lower risk of discontinuation [45]. Moreover, recent medication-taking patterns were also relevant: adherence on the previous days or during the preceding weeks, as well as consistently taking the medication at the same time each day over the course of a week, was predictive of subsequent adherence [41,45,48].

Psychosocial factors associated with higher adherence included better quality of life [39,42], improved physical, role, social, emotional, and cognitive functioning [39], and greater social support Yuan et al [48]. Moreover, patients’ knowledge, attitudes toward medicines [42], self-efficacy in managing medication [41,42], perceived susceptibility to cancer recurrence, and decision regret were identified as predictors of medication-taking behavior [41]. In contrast, baseline depression and sleep disturbance [45], as well as self-reported sadness [48], were associated with a higher predicted risk of nonadherence. Findings regarding anxiety were inconsistent: Rattsev et al [45] reported that higher baseline anxiety was associated with a lower risk of discontinuation, whereas Yuan et al [48] found that higher anxiety was associated with an increased risk of nonadherence.

Finally, among sociodemographic factors, age was the most commonly reported predictor of adherence. Nonadherence was found to be more prevalent among patients at the extremes of the age spectrum, affecting both younger (age <50 y) [43,46] and older individuals (age >65-70 y) [43,45-47]. In addition, lower income [46], being a surviving spouse, and receiving treatment in certain geographic areas [47] were also associated with nonadherence.

### Intervention Supporting Medication Adherence

Only one of the included studies described the implementation of an intervention that specifically aimed at supporting adherence by patients with BC to oral medication [40]. The authors developed an ML-based chatbot that provides personalized information via text and supports treatment management. Patients found the chatbot helpful and supportive (with an overall satisfaction rate of 93.95%) and engaged with it frequently. One of its features was a reminder function that patients could easily activate. Although 958 patients participated in the study, only 33 (3.44%) activated the reminder function for a sufficient duration to allow adherence analysis. The authors reported that these patients showed a significant increase in adherence, with a 20% increase in compliance (*P*=.04) among those who used the reminder function.

### Risk of Bias

A summary of the risk of bias and applicability assessment is presented in Table 4.

**Table 4.** Risk of bias assessment of the included prediction models using the PROBAST^a^.

First author, year					Applicability concerns			Overall	
	Participants	Predictors	Outcome	Analysis	Participants	Predictors	Outcome	RoB^b^	Applicability
Balazard et al [39], 2023	Low	Low	Low	High	Low	High	Low	High	High
Kaur et al [41], 2025	Low	Low	Low	High	Low	High	Low	High	High
Kaur et al [42], 2021	Unclear	Unclear	Low	High	Low	Unclear	Low	High	Low
Kuo et al [43], 2022	Low	Low	Low	High	Low	High	Low	High	High
Masiero et al [44], 2023	Low	Unclear	Unclear	High	Low	Low	High	High	High
Rattsev et al [45], 2024	Low	Low	Low	High	Low	High	Low	High	High
Rinder et al [46], 2024	Low	Low	Low	High	Low	High	Low	High	High
Yerrapragada et al [47], 2021	Low	Low	Low	High	Low	Low	Low	High	Low
Yuan et al [48], 2025	Low	Low	Low	High	Low	High	Low	High	High

a PROBAST: Prediction Model Risk of Bias Assessment Tool.

b RoB: Risk of Bias.

All included studies reporting on ML predictive models demonstrated a high overall risk of bias. This classification followed the PROBAST assessment criteria, according to which a high risk in any single domain results in an overall high-risk rating. In this case, the overall high-risk classification was driven by the fact that all studies showed a high risk of bias in the analysis domain. This domain includes the assessment of analytical methods and statistical considerations, as inaccuracies in applying these components may lead to bias. Specifically, the included studies do not meet the prerequisites for a comprehensive evaluation of model performance, as they do not report calibration plots or tables, nor do they assess calibration using standard methods such as the Hosmer-Lemeshow test. According to PROBAST, both discrimination and calibration must be reported when assessing a predictive model. Discrimination reflects how well a model distinguishes between cases, but without calibration, the predicted probabilities may be unreliable, potentially resulting in inappropriate clinical decisions [37,50]. Rattsev et al [45] represent an exception, as they assessed model calibration using the Integrated Brier Score. Nevertheless, this study was also judged to be at high risk of bias in the analysis domain, given the low number of events per variable, which increases the risk of overfitting.

Other studies raised applicability concerns. It is important to clarify that the applicability section of PROBAST does not refer to the general applicability of the models, but rather to their relevance to the review question defined before conducting the PROBAST assessment [37]. In this case, the review question underlying the assessment concerns ML predictive models developed to predict adherence to oral medication in patients with BC. The models developed by Masiero et al [44] raised concerns in the outcome domain, as they do not directly predict adherence. Instead, they focus on two outcomes that are reportedly associated with adherence and are used to infer potential adherence issues: (1) short- and long-term side effects, and (2) physical status and comorbid conditions. A total of 6 additional studies raised concerns in the predictors domain [39,41,43,45,46,48]. All of these included some form of prior medication-taking behavior as a predictor in their models. However, by definition, such variables are not available at the start of treatment. This raises applicability concerns if the model is intended to be used at treatment initiation, as its predictions would rely heavily on predictors that are not yet available.

Finally, it is important to note that several specific items, and consequently some domains, were rated as “unclear” due to insufficient reporting of relevant information.

The methodological quality of the only included study describing an intervention to support medication adherence [40] was assessed using Downs and Black’s methodological quality scale [38], as PROBAST is not designed for evaluating studies that do not develop or validate predictive models. As reported in Multimedia Appendix 2, the prospective study scored low across all methodological quality dimensions of the scale, indicating a high risk of bias. In particular, the low score in the “reporting” domain (5/11) was mainly due to the inadequate reporting of patient characteristics, the distribution of key confounders, and some of the study findings. The lack of information about the participants resulted in a low score (0/3) for external validity, as it was impossible to answer the scale’s items. The internal validity score (3/7) was penalized due to the inherent nature of the intervention, which made it impossible to blind participants or researchers, and because the component related to medication adherence showed very low compliance. Regarding selection bias, the low score (1/6) was primarily attributed to the absence of randomization in the study design, and once again, to the lack of relevant information about the patients and potential confounders. Finally, the power of the analysis concerning patient adherence was deemed inadequate (score: 0/5), given the small sample of participants for whom adherence could be assessed.

## Discussion

### Principal Findings

This systematic review provides a comprehensive overview of how AI-based technologies are currently being used to address medication adherence among patients with BC. Across the 10 included studies, the predominant application of AI was the development of predictive models, as all but 1 study focused on this purpose. By synthesizing their findings, we were able to identify the main groups of predictors associated with nonadherence and to report the key characteristics and performance of each model. The only study that described an intervention was also examined, and its results were summarized. This process made it possible to highlight the limitations affecting current AI applications in this area, especially in light of the thorough risk of bias assessment performed.

Drawing on the findings synthesized, several observations regarding the current landscape and suggestions for desirable future developments can be made.

To begin with, the small number of publications identified (k=10), all published between 2019 and 2025, suggests that the application of AI technologies to predict or support medication adherence is both limited and relatively recent. Nonetheless, AI-driven models appear to hold promise in predicting medication-taking behaviors, identifying key determinants of adherence, and estimating the risk of nonadherence with increasing accuracy [41-43,46,48]. However, the analysis of the findings highlights persisting limitations and unresolved challenges, underscoring the need for further research to fully unlock the potential and enhance the impact of these technologies.

### Predictive Models

We will begin by discussing the insights and observations emerging from the analyses of ML-based predictive models. First of all, the sample sizes of the included studies, except for the study by Rinder et al [46] using a sample size of 229,695, are similar to those used in predictive models using traditional statistical methods. This is a prominent point showing that AI’s potential is not yet fully realized. The ability to process large datasets is one of AI’s core strengths and could significantly enhance both the accuracy and robustness of predictive models [51,52]. A possible explanation for the sample sizes observed in the retrieved studies may lie in the need to strike an appropriate balance between sample size and the quality and relevance of the data available. Indeed, predictive models based on large datasets that include mainly irrelevant variables may still yield statistically significant results due to their size, yet prove clinically uninformative. It is crucial, therefore, not to conflate statistical significance with clinical relevance [53].

Furthermore, many of the predictors of medication nonadherence identified by AI-based models had already emerged in previous research using traditional statistical methods or qualitative approaches [10,54,55]. This convergence is to be expected, as the key contribution of AI does not lie in the discovery of new factors, but rather in enhancing analytical precision and uncovering complex interactions among multiple variables.

The predictive performance of AI models has been assessed using a range of metrics, including accuracy, sensitivity, specificity, AUC, the C-index, and the *F*_1_-score. However, performance metrics alone may not be particularly informative and should be considered alongside additional parameters to ensure the accuracy of the predictions and guarantee their clinical usefulness [56,57]. For example, 4 studies that demonstrated “excellent” performance [41-43,48] had relatively small sample sizes (ie, n=18, n=32, n=32, and n=385), which could make the models less reliable. Moreover, even the most accurate predictive model holds little value if it lacks clinical actionability, namely, if its predictions are not translated into concrete interventions, or if its implementation is not cost-effective [56]. These crucial aspects were scarcely, if at all, addressed in the studies reviewed. Considering these aspects, the performance of the models should be interpreted with caution.

According to the PROBAST evaluation, all included studies exhibited a high overall risk of bias; this result was primarily driven by a high risk of bias in the analysis domain. In fact, although the models’ discrimination performance was reported, the lack of calibration assessment exposes the predictive models to potential bias [37]. Discrimination indicates how well a model differentiates between cases, while calibration reflects how closely the predicted probabilities match the actual observed outcomes. For example, a poorly calibrated model might overestimate risk, leading to unnecessary interventions, or underestimate it, thereby missing high-risk patients who require support. This represents a major limitation, as models that are not calibrated may produce unreliable probability estimates, potentially leading to inaccurate clinical decisions when implemented in practice [50]. Underreporting of calibration in predictive model studies is a known phenomenon and has been observed across various research areas. For instance, a systematic review on predictive models for cardiovascular diseases identified that only 36% (259/717) of the studies reported calibration [58].

Another key limitation in the predictive model studies is the absence of external validation, undermining the models’ generalizability to other clinical settings, where differences in patients’ characteristics, care pathways, or data quality may significantly affect model performance. While 6 studies reported internal validation procedures, such as cross-validation or the use of a held-out validation set [39,41,45-48], none incorporated external validation. External validation involves testing the performance of the predictive model on an independent dataset to assess whether its performance is consistent and generalizable to different populations. Although this procedure could be unnecessary when large, representative samples are used, it is fundamental when models are built on smaller samples, as was the case in some of the included studies [59]. Collins et al [59] recommend that during the development of a predictive model, all available data should be used, alongside appropriate internal validation procedures. They also suggest that external validation should be conducted in subsequent studies. We were unable to identify any external validation studies for the included predictive models; however, we cannot exclude the possibility that such studies are ongoing.

Another important aspect not addressed in the included predictive model studies is implementation. The absence of reports on clinical implementation highlights a significant gap between model development and real-world application. This phenomenon is not new; the implementation gap of ML in health care has been widely acknowledged and discussed in the scientific literature [56,60]. Possible strategies to bridge this gap will be further discussed in the Clinical Implications section.

In summary, the limitations observed in the included studies describing predictive models, such as the lack of external validation, assessment of calibration, and implementation strategies, may compromise their reliability and real-world applicability. To fully realize the potential of AI in predicting medication adherence, further research is needed, with a stronger focus on methodological robustness and clinical integration.

### AI-Powered Interventions

This systematic review was designed to capture not only the role of AI in predicting patients’ medication adherence, but also its use in supporting patients and helping them develop or maintain optimal adherence. Notably, only 1 study describing an AI-powered intervention to support adherence was identified [40]. This finding is informative in itself, potentially highlighting a research area that remains underexplored and could benefit from further investigation.

Moreover, the included intervention study gives rise to several observations. Although supporting medication adherence was not the sole aim of the chatbot developed by the authors, the study reported positive results specifically related to this outcome, suggesting that AI-powered chatbots can effectively support patients with BC by helping them improve adherence. However, this result should be interpreted with caution, as only a small percentage of participants (33/958, 3.44%) used the specific reminder feature. This limited uptake raises important questions about its practical effectiveness in clinical settings and its real potential to engage patients in behavioral change. The low scores obtained in the risk of bias assessment, particularly regarding the reporting of participant characteristics, potential confounders, and the clear presentation of study findings, underscore the need to interpret the results with caution. Further research is needed to confirm the chatbot’s effectiveness, with a stronger focus on patient engagement, especially concerning the adherence support component. Future studies should ideally include a control group to better isolate the intervention’s impact on outcomes.

A final, important point to consider is the safety of this AI tool. The chatbot described in the study also provides information to patients and answers their questions. It is therefore crucial that the information delivered is accurate and reliable, as misinformation could lead to potentially dangerous and harmful consequences. Interestingly, the same chatbot was evaluated in a blinded, randomized controlled noninferiority trial, comparing the quality of the information it provided with that of physicians. The results indicated that the quality of the information provided by the chatbot was noninferior to that provided by physicians [61].

### Ethical and Safety Considerations

Ensuring safety remains a central concern in the development and use of AI-driven tools and predictive models [56]. The limited number of implementation studies makes it difficult to evaluate whether these models perform safely and reliably in real-world settings, especially when used to inform clinical decisions. This gap in evidence represents a major barrier to assessing potential risks and unintended consequences.

Privacy and data security represent equally critical issues, given that ML models rely on sensitive health information that is vulnerable to breaches and cyberattacks [62]. Recent frameworks, such as the integrated security and ethics model, have attempted to offer structured guidance by integrating principles of transparency, accountability, fairness, and privacy protection [63].

Another key challenge is algorithmic bias, often stemming from unrepresentative training data or inappropriate model generalization [20,64]. National agencies have proposed guiding principles to help stakeholders mitigate these risks, emphasizing equity, transparency, patient engagement, and accountability across the entire algorithm lifecycle [20,65].

Finally, concerns about transparency persist, as many ML models function as opaque “black boxes” [62,66]. While interpretability tools, such as Shapley values, can offer partial insight into model behavior, their use remains limited and their explanations approximate.

In summary, the ethical and safety considerations surrounding AI in health care highlight the need for responsible development and careful implementation. Adhering to established frameworks, prioritizing transparency and equity, and rigorously evaluating AI tools in real-world settings are essential steps for fostering trust and ensuring that these technologies deliver meaningful benefits in clinical practice.

### Study Limitations

This review has some limitations worth mentioning. First, although a comprehensive search strategy was used, it should be mentioned that an information scientist was not consulted during the development of the search string, and it is possible that some relevant studies were not identified. Additionally, the inclusion of only English-language publications may have resulted in the exclusion of pertinent research published in other languages.

Moreover, the recency of some included studies implies that external validation or implementation studies may still be ongoing. Their absence could limit the completeness of our evaluation, which should be revised as new evidence emerges. Nonetheless, the lack of external validation substantially impacts the reliability of the reported model performance.

In addition, the great heterogeneity of the included studies in terms of adherence measurement methods, performance metrics, variables considered, study aims, and sample sizes poses a challenge to the comparison and synthesis of their findings. Nonetheless, this variability is informative in itself, as it reflects the current state of research in this evolving field.

Another limitation to consider is the potential impact of the cancer stage on medication adherence; for example, individuals with early-stage cancer might demonstrate different motivational and psychological states compared to patients in the metastatic phase, which can influence their adherence behaviors. The heterogeneity of the population with BC included in this review (both early stage and metastatic) might limit the ability to generalize the findings across all cancer stages.

Finally, given the fast-evolving nature of the topic, this systematic review may become outdated rapidly, as AI technologies are continually advancing in power and capabilities, and new studies are constantly being conducted.

### Clinical Implications

From the comprehensive examination of the included studies, some key clinical implications can be drawn. Notably, the issue of implementation recurs as a central challenge throughout the discussion of the findings.

Investing efforts in planning, conducting, and evaluating model implementation is of paramount importance, as only through their integration into clinical practice can these models support health care professionals in identifying at-risk patients early, thereby enabling tailored interventions at an earlier stage.

It has been suggested that, in order to bridge the implementation gap, it is necessary to move beyond the excessive reliance on performance metrics such as sensitivity, specificity, and AUC. While these metrics quantify how closely predictions align with actual outcomes, they do not necessarily reflect the model’s practical utility in clinical settings. Indeed, a predictive model with high performance according to these metrics may not lead to tangible improvements in patient care [56,57]. Seneviratne et al [56] proposed 3 practical aspects that should be considered when developing a predictive model or assessing the applicability of an existing one: actionability, safety, and utility. Actionability refers to the extent to which a predictive model can inform or prompt a concrete action by the patient or the health care professional. In our context, a predictive model could be integrated into hospital clinical workflows so that, following routine assessments and screenings, if a patient is identified as being at high risk of nonadherence, an alert would automatically be triggered within their electronic medical record. This alert could then prompt appropriate interventions, such as educational initiatives, consultations with health care professionals, or referral to eHealth support programs. Safety is a key consideration in the clinical application of ML models, and it has been addressed in a previous section (see the Ethical and Safety Considerations section). Finally, utility should be carefully assessed by comparing costs and clinical outcomes with and without the implementation of the predictive model. In our context, does the use of the model lead to improved medication adherence among hospital patients? Does it reduce the costs associated with nonadherence? Is its implementation cost-effective and sustainable? These are highly relevant questions, and it is essential that clinicians and researchers consider them from the development phase and actively commit to evaluating their models against these criteria in later stages, to ensure that the potential clinical impact of the models can be fully realized.

Regarding AI-powered chatbots, further research is needed to ascertain their effectiveness in supporting medication adherence. The ethical and safety considerations discussed in the dedicated section are equally relevant to these tools, particularly in relation to patient privacy and the transparency of their underlying mechanisms [67,68]. Keeping in mind the crucial importance of meeting these prerequisites, advancements in AI technologies now make it feasible to develop AI tools that monitor and support patients throughout their cancer journey. These tools could assist patients in tracking medication-taking behaviors, deliver reminders, and guide patients in managing treatment-related side effects. Moreover, AI-powered chatbots, trained on verified clinical guidelines and scientific knowledge, can provide patients with instant support and information, reducing the burden on health care professionals [40,69]. However, for ensuring high quality of these AI models and their wide usage, it is crucial for scientific researchers, technology professionals, clinicians, and policymakers to work collaboratively to design, develop, and integrate these technologies into health care.

### Conclusions

To our knowledge, this is the first systematic review to provide a comprehensive and up-to-date overview of the current applications of AI in predicting and supporting medication adherence among patients with BC. The review encompasses both predictive and interventional studies, thereby mapping the existing applications and highlighting critical gaps. Although these technologies show potential, particularly in identifying patients at risk of nonadherence, the evidence base remains limited, and the high risk of bias observed calls for caution when interpreting current findings.

At this stage, the clinical contribution of AI remains preliminary. Meaningful progress will require stronger attention to implementation, clearer plans for real-world integration, and approaches that actively involve patients throughout development and use. Ethical and practical considerations will also need to guide each step, from development to clinical deployment. With these conditions in place, AI-based tools may, over time, evolve into a useful complement to existing adherence management strategies, but further rigorous work is needed before they can be considered ready for routine practice.

Our findings provide a foundation for future investigation and underscore the importance of a coordinated, multidisciplinary approach to enhance the accuracy, reliability, and safety of AI tools while addressing critical ethical challenges.

## Supplementary material

10.2196/80128 Multimedia Appendix 1 Search strategy.

10.2196/80128 Multimedia Appendix 2 Table for information on the prospective study.

10.2196/80128 Checklist 1 PRISMA 2020 checklist.

## Abbreviations

AI: artificial intelligence
AUC: area under the receiver operating characteristic curve
BC: breast cancer
C-index: concordance index
DL: deep learning
GenAI: generative artificial intelligence
LightGBM: light gradient boosting machine
ML: machine learning
NLP: natural language processing
PRISMA: Preferred Reporting Items for Systematic Reviews and Meta-Analyses
PRISMA-S: Preferred Reporting Items for Systematic Reviews and Meta-Analyses literature search extension
PROBAST: Prediction Model Risk of Bias Assessment Tool
PROSPERO: International Prospective Register of Systematic Reviews
SHAP: Shapley Additive Explanations
XGBoost: Extreme Gradient Boosting
