# Supplementary material for: Use of AI to Predict and Support Medication Adherence in Patients With Breast Cancer: Systematic Review
Source: JMIR Cancer. 2026 Apr 21;12:e80128. doi: 10.2196/80128 (PMC13098785; doi:10.2196/80128)
Supplement: Multimedia Appendix 1 [file cancer-v12-e80128-s001.docx]

**SCOPUS**

(TITLE-ABS-KEY(adherence) OR TITLE-ABS-KEY(compliance) OR TITLE-ABS-KEY(nonadherence) OR TITLE-ABS-KEY(non-adherence) OR TITLE-ABS-KEY(noncompliance) OR TITLE-ABS-KEY(non-compliance))

AND

(TITLE-ABS-KEY("natural language processing") OR TITLE-ABS-KEY(NLP) OR TITLE-ABS-KEY("neural networks") OR TITLE-ABS-KEY("artificial intelligence") OR TITLE-ABS-KEY(AI) OR TITLE-ABS-KEY("machine learning") OR TITLE-ABS-KEY("deep learning") OR TITLE-ABS-KEY("large language model") OR TITLE-ABS-KEY(LLM) OR TITLE-ABS-KEY(robotics))

AND

(

TITLE-ABS-KEY("breast cancer")

OR TITLE-ABS-KEY("breast neoplasm*")

OR TITLE-ABS-KEY("breast tumor*")

OR TITLE-ABS-KEY("breast tumour*")

OR TITLE-ABS-KEY("mammary cancer")

OR TITLE-ABS-KEY("mammary carcinoma*")

OR TITLE-ABS-KEY("breast carcinoma*")

)

**PUBMED**

(

((((((adherence[Title/Abstract]) OR (compliance[Title/Abstract])) OR (nonadherence[Title/Abstract]))

OR (non-adherence[Title/Abstract])) OR (noncompliance[Title/Abstract]))

OR (non-compliance[Title/Abstract]))

)

AND

(

(((((((((((("natural language processing"[Title/Abstract]) OR (NLP[Title/Abstract]))

OR ("neural networks"[Title/Abstract])) OR ("artificial intelligence"[Title/Abstract]))

OR (AI[Title/Abstract])) OR ("machine learning"[Title/Abstract]))

OR ("deep learning"[Title/Abstract])) OR ("large language model"[Title/Abstract]))

OR (LLM[Title/Abstract])) OR (robotics[Title/Abstract]))

)

AND

(

"Breast Neoplasms"[MeSH]

OR breast cancer[Title/Abstract]

OR breast neoplasm*[Title/Abstract]

OR breast carcinoma*[Title/Abstract]

OR mammary cancer[Title/Abstract]

OR mammary carcinoma*[Title/Abstract]

)

**EMBASE**

(

(((((('adherence':ti,ab,kw) OR ('compliance':ti,ab,kw)) OR ('nonadherence':ti,ab,kw))

OR ('non-adherence':ti,ab,kw)) OR ('noncompliance':ti,ab,kw))

OR ('non-compliance':ti,ab,kw))

)

AND

((((((((((('natural language processing':ti,ab,kw) OR ('nlp':ti,ab,kw))

OR ('neural networks':ti,ab,kw)) OR ('artificial intelligence':ti,ab,kw))

OR ('ai':ti,ab,kw)) OR ('machine learning':ti,ab,kw))

OR ('deep learning':ti,ab,kw)) OR ('large language model':ti,ab,kw))

OR ('llm':ti,ab,kw)) OR ('robotics':ti,ab,kw))

)

AND

(

'exp breast cancer'/exp

OR 'breast cancer':ti,ab,kw

OR 'breast neoplasm*':ti,ab,kw

OR 'breast carcinoma*':ti,ab,kw

OR 'mammary cancer':ti,ab,kw

OR 'mammary carcinoma*':ti,ab,kw

)

**WEB OF SCIENCE**

TS=(

adherence OR compliance OR nonadherence OR non-adherence OR noncompliance OR non-compliance

)

AND

TS=(

"natural language processing" OR NLP OR "neural networks"

OR "artificial intelligence" OR AI

OR "machine learning" OR "deep learning"

OR "large language model" OR LLM

OR robotics

)

AND

TS=(

"breast cancer"

OR "breast neoplasm*"

OR "breast tumor*" OR "breast tumour*"

OR "breast carcinoma*"

OR "mammary cancer"

OR "mammary carcinoma*"

)
