# Supplementary material for: Use of AI to Predict and Support Medication Adherence in Patients With Breast Cancer: Systematic Review
Source: JMIR Cancer. 2026 Apr 21;12:e80128. doi: 10.2196/80128 (PMC13098785; doi:10.2196/80128)
Supplement: Multimedia Appendix 2 [file cancer-v12-e80128-s002.docx]

**Table 5.**Methodological quality assessment of the intervention study using Downs and Black’s scale.

| **First author, year** | **RB^a^ (0‐11)** | **EV^b^ (0‐3)** | **IV^c^ (0‐7)** | **SB^d^ (0‐6)** | **P^e^ (0‐5)** | **Total (0‐32)** |
| --- | --- | --- | --- | --- | --- | --- |
| Chaix et al [40], 2019 | 5 | 0 | 3 | 1 | 0 | 9 |

**Notes:** ^a^RB: reporting bias.^b^EV: external validity.^c^IV: internal validity.^d^SB: selection bias.^e^P: power.
